# Factors Associated With Body Image Distress in Patients With Head and Neck Cancer: Protocol for a Systematic Review

**DOI:** 10.2196/69213

**Published:** 2025-10-15

**Authors:** Wenjie Xu, Lina Xiang, Shuman Wang, Mimi Zheng, Rong Ge, Yu Zhu, Hongwei Wan

**Affiliations:** 1 Department of Nursing, Shanghai Proton and Heavy Ion Center, Fudan University Cancer Hospital, 4365 Kangxin Rd, Pudong New Area, Shanghai, China, 86 13459339083; 2 Shanghai Key Laboratory of Radiation Oncology, Shanghai, China; 3 Shanghai Engineering Research Center of Proton and Heavy Ion Radiation Therapy, Shanghai, China; 4 Department of Nursing Mental Health Center, Shanghai Jiao Tong University School of Medicine, Shanghai, China; 5 School of Nursing, Shanghai Jiao Tong University, Shanghai, China; 6 Department of Nursing, Zhongshan Hospital, Fudan University, Shanghai, China; 7 Department of Nursing, Ruijin Hospital, Shanghai Jiaotong University School of Medicine, Shanghai, China

**Keywords:** body image distress, head and neck cancer, systematic review, psychosocial factors, quality of life

## Abstract

**Background:**

Body image distress (BID) is a significant psychological issue for patients with head and neck cancer (HNC) and arises from the visible disfigurements and functional impairments often associated with the disease and its treatment. Understanding the factors contributing to BID in this population is crucial for developing effective interventions and support mechanisms.

**Objective:**

This systematic review outlines methods for identifying, evaluating, and synthesizing the available evidence on factors associated with BID among patients with HNC. This review intends to explore both clinical and psychosocial variables that may influence body image perceptions and the resulting psychological impact.

**Methods:**

This review will follow the Joanna Briggs Institute (JBI) Reviewer’s Manual for Systematic Reviews Concerning Etiology and Risk Factors. PsycINFO, MEDLINE, EBSCOhost CINAHL, Web of Science, Cochrane Library, and Embase were searched for relevant studies from inception to December 2024. All in-depth quantitative analyses, descriptive observational studies, experimental studies, and quasi-experiments published in English or Chinese were analyzed and described. Studies examining factors associated with BID in patients with HNC were included. The population, exposure, comparison, and outcome (PECO) format was used to develop the search strategy. For different databases, search terms will be combined using Boolean operators. The JBI Risk of Bias Tool was used to evaluate bias risk in the included studies. The extracted data will include basic study information, research design, sample characteristics, BID measurement tools, primary outcomes, statistical analysis methods, and quality assessment results. Subgroup and meta-regression analyses will be performed on different therapies, treatment stages, genders, ages, cultural backgrounds, etc. *I*^2^ statistics will be used to evaluate heterogeneity, and funnel plots will address publication bias. If we detect significant heterogeneity, the findings will be reported as a systematic review without a meta-analysis.

**Results:**

The database search will be conducted in October 2025. It is anticipated that the study findings will be submitted for publication in a peer-reviewed journal by the end of March 2026.

**Conclusions:**

This study will summarize the factors that can help identify and evaluate the factors associated with BID in patients with HNC, providing up-to-date evidence to inform the management of body image in this patient population.

## Introduction

### Background

Head and neck cancer (HNC), a diverse group of malignancies affecting the oral cavity, pharynx, larynx, and more, ranks as the sixth most common cancer type worldwide, leading to more than 600,000 new cases annually [1]. Survival rates are approximately 40%‐50%, and early detection and comprehensive treatment strategies, including surgery, radiation therapy, and chemotherapy, are crucial. However, surgery and radiation therapy, despite their effectiveness, can lead to severe side effects. The most common acute side effect is mucositis, which can cause significant pain and impact a patient’s ability to eat and speak [2]. Other acute side effects include xerostomia (dry mouth), dysphagia (difficulty swallowing), and odynophagia (painful swallowing). Long-term side effects can include fibrosis, lymphedema, and osteoradionecrosis. Among these, skin reactions are prevalent and can be particularly distressing for patients because they are often visible and disfiguring [3]. These skin reactions can range from mild erythema to severe ulceration and necrosis, leading to significant body image distress (BID) [4].

BID is defined as a negative self-perception of one’s body arising from a discrepancy between one’s actual physical appearance and ideal body image [5]. It encompasses a range of emotional, cognitive, and behavioral disturbances that can affect an individual’s self-esteem, mood, and social functioning. For patients with HNC, the impact of BID can be profound. Given the visible changes in appearance and function and their impact on daily social interactions, it is not surprising that 75% of HNC survivors express concerns about their body image [6], with up to 28% experiencing clinically significant BID [7,8]. The visible disfigurements and functional impairments resulting from the cancer itself, as well as from treatments such as surgery and radiation therapy, can lead to significant distress. Patients may experience feelings of self-consciousness, shame, and social isolation as a result of changes in their appearance and abilities [9-11]. These psychosocial challenges can exacerbate the physical difficulties faced during treatment and recovery, potentially leading to depression, anxiety, and a diminished quality of life [12,13].

The literature on factors influencing BID in patients with HNC is expanding, with numerous studies examining a variety of potential contributors. These factors can be broadly categorized into clinical variables, such as tumor location, stage, and treatment side effects, and demographic variables, such as age, sex, and cultural background [12,14]. Research has also begun to explore the role of psychosocial variables, including social support, coping strategies, and preexisting mental health conditions, in influencing the experience of BID [15]. However, most evidence comes from small, single-center cross-sectional surveys that use heterogeneous instruments and lack multivariable adjustment. Consequently, the magnitude and interplay of these putative determinants remain poorly quantified, and no integrated framework currently exists. To address this gap, we conducted the first systematic review and meta-analysis to quantitatively appraise and synthesise the clinical, demographic, and psychosocial factors associated with BID in HNC survivors.

Exploring the factors influencing BID in patients with HNC is crucial and indispensable for developing targeted psychosocial interventions. Existing research on HNC-related BID has predominantly used a cross-sectional design, yielding heterogeneous results that reflect the complexity of body image issues in this patient population. For instance, Fingeret et al [16] found that individuals with higher levels of education may experience lower levels of BID. Conversely, Jiayan et al [17] reported that higher educational levels are associated with more severe BID, possibly due to the higher social and economic status of these patients and their greater demands for self-image. This discrepancy underscores the need for more longitudinal and comprehensive studies to better understand the multifaceted nature of BID in the context of HNC. Moreover, the psychological aspects of body image, which are multifactorial and encompass elements such as self-esteem, depression, and social support, are also critical to address, as highlighted by Patterson et al [18]. These studies indicate the complexity of the factors at play and the need for a more nuanced understanding of the psychosocial needs of patients with HNC.

The purpose of this systematic review was to summarize the literature on the factors associated with BID in patients with HNC. By identifying these factors, this review aims to inform health care professionals and researchers about the key areas targeted for psychosocial support and intervention. Furthermore, this study seeks to identify gaps in the current research, providing a roadmap for future studies to enhance our understanding of BID in patients with HNC and to improve their overall care and quality of life.

### Research Questions

This review seeks to answer the following two research questions: What is the level of BID in patients with HNC? How do the individual characteristics of patients with HNC (including age, sex, social support status, and mental health status) affect the level of BID?

## Methods

### Standards

The methodological framework for this systematic review is grounded in the guidelines outlined in the Joanna Briggs Institute (JBI) Reviewer’s Manual for Systematic Reviews Concerning Etiology and Risk Factors [19]. Additionally, the methodology is informed by the processes described by Dekkers et al [20]. This review protocol strictly conforms to the guidelines set forth by the PRISMA-P (Preferred Reporting Items for Systematic Reviews and Meta-Analysis Protocols) statement [21]. Furthermore, the final systematic review will comply with the PRISMA (Preferred Reporting Items for Systematic Reviews and Meta-Analyses) guidelines [22]. To ensure transparency and reproducibility, this protocol has been proactively registered in PROSPERO (unique registration number CRD42023424006).

### The Eligibility Criteria

English- and Chinese-language reports published in peer-reviewed sources were included.

### Types of Studies

These studies include original observational studies, such as case-control, cohort, and cross-sectional studies, demonstrating a quantitative relationship between at least one associated factor and HNC-related BID. Experimental studies and quasi-experiments will also be included, although these are less likely to assess factors associated with BID. We excluded conference abstracts, qualitative articles, commentaries, letters to the editor, books, systematic reviews, meta-analyses, and case reports. There will be no restrictions based on the type of setting. Studies published in English or Chinese are considered. There will be no restrictions on the year of publication.

### Types of Participants

Studies involving patients with HNC diagnosed regardless of sex, race, or educational background will be included. Studies included individuals with a confirmed HNC diagnosis, as evidenced by medical records, histopathological confirmation, and diagnostic criteria, as specified in the study. All studies must be inclusive of any gender identities. Studies exclusively focusing on one gender, unless providing a justified scientific rationale, will be excluded to ensure a diverse and comprehensive understanding of BID across the population. Studies should include adult patients (aged 18 years and older) with HNC. If age-specific factors related to BID are of interest, age subgroups (eg, young adults, middle-aged, and older adults) should be specified in the protocol.

### Conditions

BID, as the variable of interest in our systematic review protocol, refers to the psychological discomfort or distress that a patient with HNC experiences due to changes or perceived changes in their appearance. To be included in our review, studies had to measure BID either as a primary or secondary outcome. BID will be quantified using validated psychometric tools specifically designed to assess body image concerns in patients with cancer, such as the Derriford Appearance Scale (DAS-24), the Inventory to Measure and Assess imaGe disturbance-Head and Neck (IMAGE-HN), the Body Image Scale (BIS), and the McGill Body Image Concerns Scale-Head and Neck Cancer (MBIS-HNC). Studies included in the review will provide clear information on the threshold used to define clinically significant BID, ensuring that only patients who meet or exceed this threshold are considered. Our systematic review will, therefore, encompass articles that quantify BID using these standardized tools, allowing us to synthesize data on how frequently this distress occurs and identify factors associated with increased BID.

### Context

We will include studies that evaluate factors associated with HNC-related BID, covering a range of demographic, clinical, and psychosocial variables such as age, sex, type and extent of surgery, level of education, stage of cancer, presence of anxiety or depression, availability and quality of social support, and coping styles. Additionally, we will consider the role of treatment modalities (eg, chemotherapy and radiation therapy), physical side effects (eg, disfigurement and functional impairment), and patient-reported outcomes related to quality of life. Studies that provide a comprehensive analysis of the relationships between these variables and BID—using quantitative, qualitative, or mixed methods approaches—will be included.

### Information Sources

**Figure 1.**
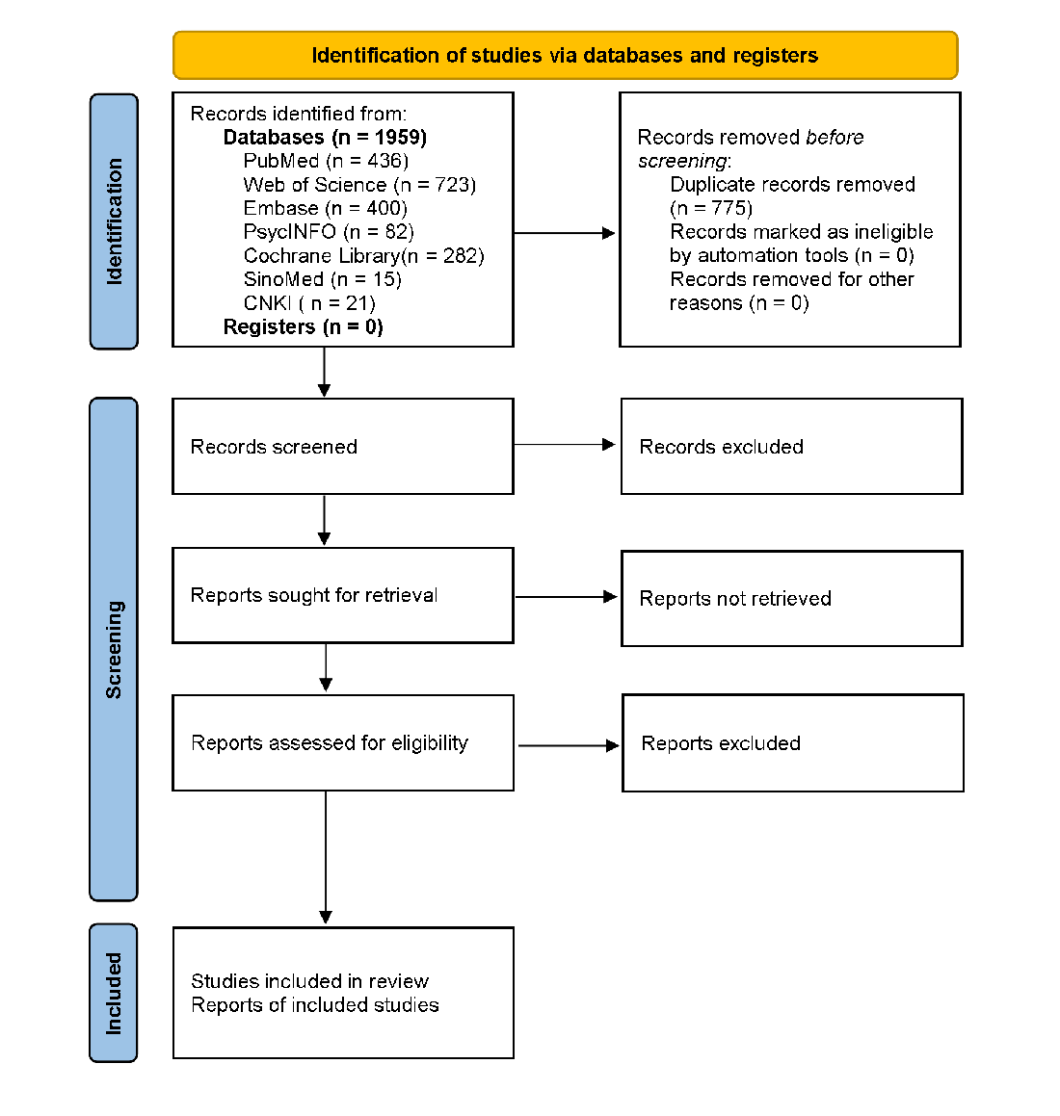
PRISMA (Preferred Reporting Items for Systematic Reviews and Meta-Analyses) flow diagram of the screening process.

PsycINFO, MEDLINE, EBSCOhost CINAHL, Web of Science, Cochrane Library, and Embase will be comprehensively searched from inception to October 2025. The preliminary search process is shown in Figure 1. An initial search using this filter yielded several authors (WX, LX, SW, MZ, and YZ). Additionally, we will contact the authors of the articles identified for inclusion in our systematic review and possible meta-analyses regarding the details of the completed but unpublished research. We will locate ongoing clinical trials by searching prospective clinical trial registries (national and international).

### Search Strategy

The population, exposure, comparison, and outcome (PECO) format was used to develop the search strategy. The most important keywords for each PECO component are as follows.

#### Comparison

The search strategies were developed using a combination of keywords and standardized index terms. The literature search will be limited to articles written in English or Chinese and involving human participants. To ensure comprehensive coverage, we scanned the reference lists of the included studies and relevant reviews identified through the search. Additionally, we will search the authors’ files to ensure that all relevant material has been included. Finally, the systematic review team will circulate a bibliography of the included articles. There will be no time restrictions for the search.

#### Population

Patients with HNC who are older than 18 years; have no serious heart, brain, kidney, or other functional failure diseases; have no consciousness disorders; and have not received psychological treatment will comprise the study population.

#### Exposure

The factors considered include personal factors (such as sociodemographic and psychological factors), disease-related factors, and sociocultural background factors.

#### Outcome

BID and stress related to body image are the outcomes of interest.

A preliminary literature search was conducted by a health sciences librarian specializing in systematic reviews, with input from the study investigators. The draft MEDLINE search strategy can be found in Multimedia Appendix 1. Once the MEDLINE strategy is finalized, it will be adapted to match the syntax and subject headings of the other databases. As relevant studies are identified, reviewers will also search for additional relevant articles that have been cited or those cited by the identified studies. Additionally, the search will be updated toward the end of the review to ensure that the PubMed strategy retrieves a high proportion of eligible studies found through any means but indexed in PubMed.

### Assessment of Methodological Quality

The systematic review protocol will specify the criteria used to determine the methodological quality of the papers included in the review. In line with these requirements, the JBI critical appraisal tool will be used preferentially for assessing the quality of studies [23]. These tools are chosen for their comprehensive nature and their ability to evaluate a wide range of study designs and methodologies.

### Criteria for Methodological Quality

The criteria for assessing methodological quality will include, but will not be limited to, the following: study design appropriateness, clarity of objectives, use of valid and reliable measures, adequacy of sample size, handling of data and analysis, consideration of confounders, and robustness of the findings. These criteria ensure that only studies with sufficient methodological rigor are included in the review.

### Use of JBI Tools

For each type of study design identified in the search process, the corresponding JBI critical appraisal checklist will be used. The JBI Checklist for Analytical Cross-Sectional Studies will be used for observational studies [24].

### Critical Appraisal for Prevalence Studies

Given the possibility of prevalence data emerging from various study designs, including RCTs, a specific critical appraisal checklist for prevalence studies has been developed by JBI. This checklist will be used to ensure a consistent and targeted approach to appraising studies reporting prevalence data.

### Independent Review Process

Two independent reviewers (WX and SW) will critically appraise each study. The reviewers will use the designated JBI critical appraisal tools to systematically assess and document the methodological quality of the studies. They will work independently to minimize bias and enhance the reliability of the appraisal process.

### Consensus and Dispute Resolution

Upon completion of the independent appraisals, the reviewers will convene to discuss their findings and reach a consensus on the final appraisal for each study. This collaborative approach fosters transparency and agreement in the assessment of methodological quality. When 2 reviewers cannot reach a consensus through discussion, a third reviewer will be called to provide an independent evaluation to resolve the disagreement.

By meticulously detailing the criteria and process for methodological quality assessment, this protocol ensures that the systematic review will be based on rigorously appraised evidence. The independent and collaborative approach to critical appraisal enhances the validity of the review findings and supports the integrity of the review process.

### Data Extraction

The authors (WX and SW) independently screened the titles and abstracts against the inclusion criteria. Reviewers will submit a detailed report if there is any uncertainty or if a title appears to meet the inclusion criteria. Reviewers will then screen the full-text reports to determine if they comply with the inclusion criteria. If an article meets the inclusion criteria but is not available through our institution’s library access, the authors of the article will be contacted via email to obtain the full text. Reviewers will record the reasons for excluding trials. The review authors will not be blinded to the journal titles, study authors, or institutions.

After the final number of studies included in the systematic review is determined, the data will be extracted. The data extraction tool will be developed specifically for this review, and a data collection form will be created using Microsoft Excel 2020. The tool will be tested on 2‐4 articles and modified as needed before being used. WX and SW will independently extract data from each study. We will extract the following information from each study: study design, sample size, inclusion/exclusion criteria, evaluated risk factors, and outcome measures (ie, BID assessment tool, presence/absence of BID, the overall frequency of BID, raw numbers of actors associated with patients with and those without HNC related-BID, and results from univariate and multivariate analyses). To extract effect measures (both ratio and difference measures), we will follow the recommendations of the JBI. Both unadjusted and adjusted estimates will be extracted. In the analysis of factors associated with BID, confounding factors will be considered, and the list of confounders will be collected for each study. We will contact the authors via email if the necessary data are not included in the published manuscript.

### Data Synthesis

To investigate the associations between factors and the burden of BID in patients with HNC, we intend to conduct a meta-analysis. This analysis will commence after evaluating the data availability and extent of heterogeneity across the included studies. If the studies demonstrate sufficient homogeneity, we will compute pooled odds ratios for each factor by using a fixed/random effects meta-analysis model. The choice to use a fixed/random-effects model will be based on the level of heterogeneity both within and across the studies. We will measure statistical heterogeneity and inconsistency by estimating the *I*^2^ index. The analysis will be conducted using RevMan (version 5.3; The Cochrane collaboration) and MetaXL software (EpiGear International Pty Ltd), and we will derive meta-analytic estimates for both unadjusted and adjusted data separately.

### Ethical Considerations

Ethics approval will not be necessary because the publications included in our study did not involve patient privacy. The main data will be extracted from published literature. This systematic review will be published in a peer-reviewed journal with significant visibility in the field of psychosocial oncology.

To discern potential confounding factors, we will compare both adjusted and unadjusted estimates. A meta-analysis of factors associated with BID may present challenges due to considerable methodological heterogeneity among the studies. As an alternative approach to synthesizing data on factors associated with BID, we will use harvest plots, which are especially advantageous for addressing broader research questions.

We also provide a methodology for synthesizing results when studies report only the direction of an association or when there is inconsistency in the measured effects across studies. Subgroup analyses will be conducted to explore differences between patients with and without BID, to examine study design factors such as retrospective versus prospective observational studies, and to evaluate study quality, specifically the risk of bias ranging from low to high.

### Synthesizing Evidence

We will apply the GRADE (Grading of Recommendations, Assessment, Development, and Evaluations) approach to grade the quality of evidence, considering factors such as imprecision, inconsistency, indirectness, and publication bias. The most robust evidence for prognostic factors or those related to the topic may initially be assigned high certainty ratings [25]. Additionally, we will consider other domains, including the magnitude of the effect and the potential impact of residual confounding [26]. For example, a substantial effect estimate (ie, relative effect size over 2.0) may warrant an upgrade in the certainty level. Depending on the concerns related to these domains, evidence certainty will ultimately be classified as high, moderate, low, or very low [25]. To create evidence summaries, we will use GRADEpro software via the GRADEpro GDT website. We will assess the evidence quality (certainty in estimates) for each factor associated with HNC-related BID. In cases where significant heterogeneity across studies prevents us from conducting a meta-analysis, we will adhere to the framework proposed by Murad et al [27] for determining evidence certainty without a single effect estimate.

## Results

At the time of writing, the literature search had been completed by the medical librarian, and the screening of titles and abstracts is in progress. The database search will be conducted in October 2025. We anticipate completing the screening process by December 2025 and submitting the manuscript for peer review by the end of March 2026. The research findings will be submitted to journals specializing in head and neck tumors or body image, presented at relevant conferences, and shared with stakeholders, including nongovernmental organizations, educational and health care institutions, assistive technology developers, and policy makers.

## Discussion

In conclusion, this systematic review protocol outlined a structured approach to identify and evaluate the factors associated with BID in patients with HNC. By conducting a comprehensive meta-analysis, we aim to synthesize existing data to better understand the extent to which various demographic, clinical, and psychosocial factors contribute to BID in this population. The use of both adjusted and unadjusted meta-analytic estimates will allow for a nuanced examination of potential confounding variables.

The anticipated challenges in comparing factors across studies due to methodological heterogeneity will be addressed by using alternative methods, such as harvest plots for data synthesis. Subgroup analyses will further refine our understanding by comparing different patient groups, study designs, and levels of study quality. Although publication bias remains a concern, our commitment to analyzing only those estimates supported by a sufficient number of studies should mitigate this issue.

The possible strengths and limitations of this study are as follows. The main strength of this systematic review is its novelty. This study will help identify iatrogenic risk factors for HNC-related BID, which may be helpful in developing future interventions. The findings of this study will inform researchers of the future development of clinical prediction rules when identifying patients with HNC at risk for BID. Some studies might need a more well-defined differentiation of BID. The significant heterogeneity across studies may prevent us from performing meta-analyses. The quality of the included studies is expected to be low, resulting in a low level of certainty regarding the associations between certain factors and HNC-related BID. The types of studies included were mostly cross-sectional, and more high-quality longitudinal studies analyzing the factors influencing BID in patients with HNC are needed. Additionally, the review is limited to English- and Chinese-language publications, potentially excluding relevant evidence in other languages, and qualitative studies that could offer deeper insights into patient experiences are omitted.

## Supplementary material

10.2196/69213 Multimedia Appendix 1 Draft of the MEDLINE search query.

## Abbreviations

BID: body image distress
BIS: Body Image Scale
DAS-24: Derriford Appearance Scale
GRADE: Grading of Recommendations, Assessment, Development, and Evaluations
HNC: head and neck cancer
IMAGE-HN: Inventory to Measure and Assess imaGe disturbance-Head and Neck
JBI: Joanna Briggs Institute
MBIS-HNC: McGill Body Image Concerns Scale-Head and Neck Cancer
PECO: population, exposure, comparison, and outcome
PRISMA: Preferred Reporting Items for Systematic Reviews and Meta-Analyses
PRISMA-P: Preferred Rpeorting Items for Systematic Reviews and Meta-Analyses Protocols
