# Supplementary material for: Factors Associated With Body Image Distress in Patients With Head and Neck Cancer: Protocol for a Systematic Review
Source: JMIR Res Protoc. 2025 Oct 15;14:e69213. doi: 10.2196/69213 (PMC12527319; doi:10.2196/69213)
Supplement: Multimedia Appendix 1 [file resprot-v14-e69213-s001.docx]

**Appendix 1**

**Draft MEDLINE search (Interface: PubMed)**

#1: (("Head and Neck Neoplasms"[Mesh]) OR (Neoplasms, Head and Neck[Title/Abstract) OR (Head, Neck Neoplasms[Title/Abstract) OR (Head and Neck Neoplasm[Title/Abstract) OR (Cancer of Head and Neck[Title/Abstract) OR (Head and Neck Cancer[Title/Abstract) OR (Cancer of the Head and Neck[Title/Abstract) OR (Upper Aerodigestive Tract Neoplasms[Title/Abstract) OR (UADT Neoplasm[Title/Abstract) OR (Neoplasm, UADT[Title/Abstract) OR (Neoplasms, UADT[Title/Abstract) OR (UADT Neoplasms[Title/Abstract) OR (Neoplasms, Upper Aerodigestive Tract[Title/Abstract) OR (Upper Aerodigestive Tract Neoplasm[Title/Abstract) OR (Head Neoplasms[Title/Abstract) OR (Neoplasms, Head[Title/Abstract) OR (Head Neoplasm[Title/Abstract) OR (Neoplasm, Head[Title/Abstract) OR (Neck Neoplasms[Title/Abstract) OR (Neoplasms, Neck[Title/Abstract) OR (Neck Neoplasm[Title/Abstract) OR (Neoplasm, Neck[Title/Abstract) OR (Cancer of Head[Title/Abstract) OR (Head Cancers[Title/Abstract) OR (Head Cancer[Title/Abstract) OR (Cancer, Head[Title/Abstract) OR (Cancers, Head[Title/Abstract) OR (Cancer of the Head[Title/Abstract) OR (Cancer of Neck[Title/Abstract) OR (Neck Cancers[Title/Abstract) OR (Neck Cancer[Title/Abstract) OR (Cancer, Neck[Title/Abstract) OR (Cancers, Neck[Title/Abstract) OR (Cancer of the Neck[Title/Abstract))

#2: (("Body Image"[Mesh]) OR (Body Images[Title/Abstract) OR (Image, Body[Title/Abstract) OR (Body Identity[Title/Abstract) OR (Identity, Body[Title/Abstract) OR (Body Representation[Title/Abstract) OR (Body Representations[Title/Abstract) OR (Representation, Body[Title/Abstract) OR (Body Schema[Title/Abstract) OR (Body Schemas[Title/Abstract) OR (Schema, Body[Title/Abstract))

#3: ((Risk Factor[Mesh]) OR (Factor, Risk[Title/Abstract) OR (Risk Factor[Title/Abstract) OR (Social Risk Factors[Title/Abstract) OR (Factor, Social Risk[Title/Abstract) OR (Factors, Social Risk[Title/Abstract) OR (Risk Factor, Social[Title/Abstract) OR (Risk Factors, Social[Title/Abstract) OR (Social Risk Factor[Title/Abstract) OR (Health Correlates[Title/Abstract) OR (Correlates, Health[Title/Abstract) OR (Population at Risk[Title/Abstract) OR (Populations at Risk[Title/Abstract) OR (Risk Scores[Title/Abstract) OR (Risk Score[Title/Abstract) OR (Score, Risk[Title/Abstract) OR (Risk Factor Scores[Title/Abstract) OR (Risk Factor Score[Title/Abstract) OR (Score, Risk Factor[Title/Abstract) OR ("Dangerous Factor"[Title/Abstract) OR ("Relat* Factor"[Title/Abstract) OR ("Relevant Factor"[Title/Abstract) OR ("Factors Associated"[Title/Abstract) OR (risk*[Title/Abstract) OR (predict*[Title/Abstract) OR (associat*[Title/Abstract) OR (correlat*[Title/Abstract) OR (model*[Title/Abstract) OR (predispos*[Title/Abstract) OR (susceptib*[Title/Abstract) OR [Title/Abstract))

#4: #1 AND #2 AND #3
